# Leveraging Experience From Active TB Drug-Safety Monitoring and Management for Monitoring Active Antiretroviral Toxicity

**DOI:** 10.9745/GHSP-D-21-00595

**Published:** 2022-04-28

**Authors:** Lisa Stevens, Kelly E. Perry, Iakuna Moide, Francil Kaemala, Justine Nankinga, Anh L. Innes, Ignatius Mogaba

**Affiliations:** a FHI 360 Asia Pacific Regional Office, Bangkok, Thailand.; b FHI 360 Papua New Guinea Office, Port Moresby, Papua New Guinea.; c FHI 360 Vietnam Office, Hanoi, Vietnam.

## Abstract

Systems established for active drug safety monitoring and management of drug-resistant TB should be leveraged to ensure comprehensive surveillance for active toxicity monitoring during scale-up of newer antiretroviral regimens.

## Key Messages

- The introduction of novel medicines and regimens for antiretroviral (ARV) and TB treatment requires comprehensive surveillance systems for adverse events and adverse drug reactions.
- Many TB programs have introduced and institutionalized active drug safety monitoring and management (aDSM) platforms for drug-resistant TB.
- Because HIV programs must develop active ARV toxicity monitoring systems to ensure safe global scale-up of newer regimens, such as tenofovir-lamivudine-dolutegravir, we propose building on existing aDSM infrastructure to actively monitor ARV regimens as a synergistic TB/HIV collaborative activity and to narrow active toxicity monitoring gaps.

## INTRODUCTION

TB and HIV are preventable diseases, yet both continue to cause significant suffering and loss of life worldwide. Although concerted efforts have led to declining TB and HIV incidence and mortality, much remains to be done to reach global goals.^1^^,^^2^ Countries strive to reach “95-95-95” goals established by the Joint United Nations Programme on HIV/AIDS (UNAIDS), which include 95% of people living with HIV (PLHIV) knowing their status, 95% of those known to have HIV taking antiretroviral therapy (ART), and 95% of PLHIV on ART achieving viral suppression.^3^ Global TB programs endeavor to achieve the END TB Strategy goals set by the World Health Organization (WHO), including 95% global reduction in deaths due to TB and 90% reduction in TB incidence by 2035 compared to 2015.^4^

Although declining incidence is multifactorial and strategies differ between TB and HIV programming, one critical contributor to reduced incidence and mortality has been the development of new medicines and better treatment regimen options, especially for HIV and multidrug-resistant (MDR) TB.

For many years after initial ART introduction in resource-limited settings, programs relied on regimens containing either zidovudine or stavudine. Zidovudine is known to cause myelosuppression, with a study in northeastern Nigeria showing that anemia developed in 22.3% of those taking the drug.^5^^,^^6^ Similarly, stavudine can induce a range of metabolic and long-term complications, with more than 90% of those taking stavudine in Cambodia requiring a switch to an alternative within 6 years of initiating therapy due to toxicity,^7^ mainly lipoatrophy.^8^

International guidelines now recommend first-line HIV drugs with better side effect profiles than “older” drugs such as zidovudine and stavudine.^9^ The introduction of new drugs and regimens is critical to attaining global HIV goals through treatment optimization with better-tolerated products. Although the overall aim is to improve tolerability, during widespread global scale-up, adverse events may emerge, some of which may be predictable, mild, and reversible while others could be unexpected or more serious. Strong surveillance systems are needed to proactively detect and monitor adverse events at individual, national, and global levels.

Most countries have existing pharmacovigilance systems established for reporting adverse drug reactions (ADRs) at the national level. However, in many low- and middle-income countries (LMICs), such systems are not well functioning or operate only through passive reporting, often requiring patients themselves to inform health care providers of adverse events that might be related to a medication.^10^^,^^11^ Health systems often lack a systematic approach to actively monitor patients for ADRs. Thus, critical signals can be missed. With current global transitions—including a shift from efavirenz-based ART regimens to those containing dolutegravir,^9^ availability of pediatric dispersible dolutegravir formulation for young children and infants,^12^ and the anticipated introduction of long-acting antiretrovirals (ARVs)^13^—it is imperative that providers and programs are vigilant in actively monitoring and managing ADRs from ARVs with sustainable reporting systems established.

Meanwhile, in TB programs, the WHO was recommending an injectable aminoglycoside- containing regimen among MDR-TB treatment options as late as May 2019. These agents induce ototoxicity manifesting as vestibulotoxicity in up to 15% of patients and cochleotoxicity in 2% to 25% of patients, often resulting in permanent hearing loss.^14^ The WHO now recommends the use of the new all-oral MDR-TB regimens, which have recently become the standard of care in many countries, yet alarmingly almost half of the 37 high-TB burden countries surveyed in the *Step Up for TB Survey* in 2020 continued to use the more toxic injectable medicines.^15^

Although treatment for drug-susceptible TB has remained unchanged since the 1980s,^16^ shorter potent regimens are expected to be introduced in the near future, following the recently published TBTC Study 31/ACTG A5349 trial results, which demonstrated noninferiority of a novel 4-month anti-TB drug regimen compared to the standard 6-month treatment.^17^

Many TB programs have introduced and institutionalized active drug safety monitoring and management (aDSM) platforms for drug-resistant TB to support the recent introduction of both new drugs and novel combination regimens. These have been instrumental in the shift away from injectable-based MDR-TB treatment to all-oral regimens, which still have potential toxicities that require monitoring.^18^ Leveraging previous efforts within TB programs to establish aDSM and strengthen pharmacovigilance can provide a foundation to initiate similar system strengthening focused on improved HIV treatment regimens.

## A BRIEF OVERVIEW OF ADSM FOR TB

MDR-TB treatment has evolved since the WHO introduced the DOTS PLUS concept in 2000.^19^ The WHO estimated that 465,000 people had MDR-TB globally in 2019, yet the current MDR-TB treatment success rate was only 57%.^2^ In addition, 1,350 incident cases of extensively drug-resistant (XDR) TB were reported, where mycobacteria are resistant not only to rifampicin and isoniazid as in MDR-TB but also to both a fluoroquinolone and at least 1 second-line injectable agent.^2^ To combat both MDR- and XDR-TB, new drugs and regimens have been developed and are at various stages of research and approval. The U.S. Agency for International Development (USAID) and Janssen Therapeutics together accelerated access to one of these game-changing medications, bedaquiline, through a 4-year global public-private partnership in more than 100 countries.^20^ Due to the poor global treatment success rate and the urgent need for better options, new drugs and regimens for MDR/XDR-TB are often used off-label or before completion of phase 3 trials, rendering monitoring and reporting of ADRs even more critical.

New TB drugs and regimens are often used off-label or before completion of phase 3 trials, rendering monitoring and reporting of ADRs even more critical.

The WHO's treatment recommendations currently range from 9 to 20 months of daily combination treatment, depending on the drug-resistance profile and availability of medications in-country. Second-line TB medicines are frequently associated with adverse events, ranging from mild to life-threatening, and often lead to regimen change.^21^ Due to toxicity profiles and efficacy issues, elimination of injectable agents is now recommended across all countries.^18^ However, all-oral regimens also contain potentially toxic medications and need to be carefully monitored and managed.

Specific to TB, aDSM systems are currently being established and implemented to ensure robust and systematic clinical and laboratory assessment of patients while on TB treatment. The concept of aDSM was developed by the TB community to better manage treatment with new TB drugs and novel MDR- or XDR-TB regimens to more effectively and efficiently detect, manage, and report suspected or confirmed drug toxicities. The WHO defines aDSM as^22^:

*active and systematic clinical and laboratory assessment of people being treated for drug-resistant TB with new TB medicines or novel MDR-TB regimens to detect, manage, and report suspected or confirmed drug toxicities.*

aDSM is also a useful tool for programmatic management of drug-resistant TB, which allows care providers to rapidly adjust regimens, as needed.^23^

### aDSM Components and Processes

**FIGURE 1:**
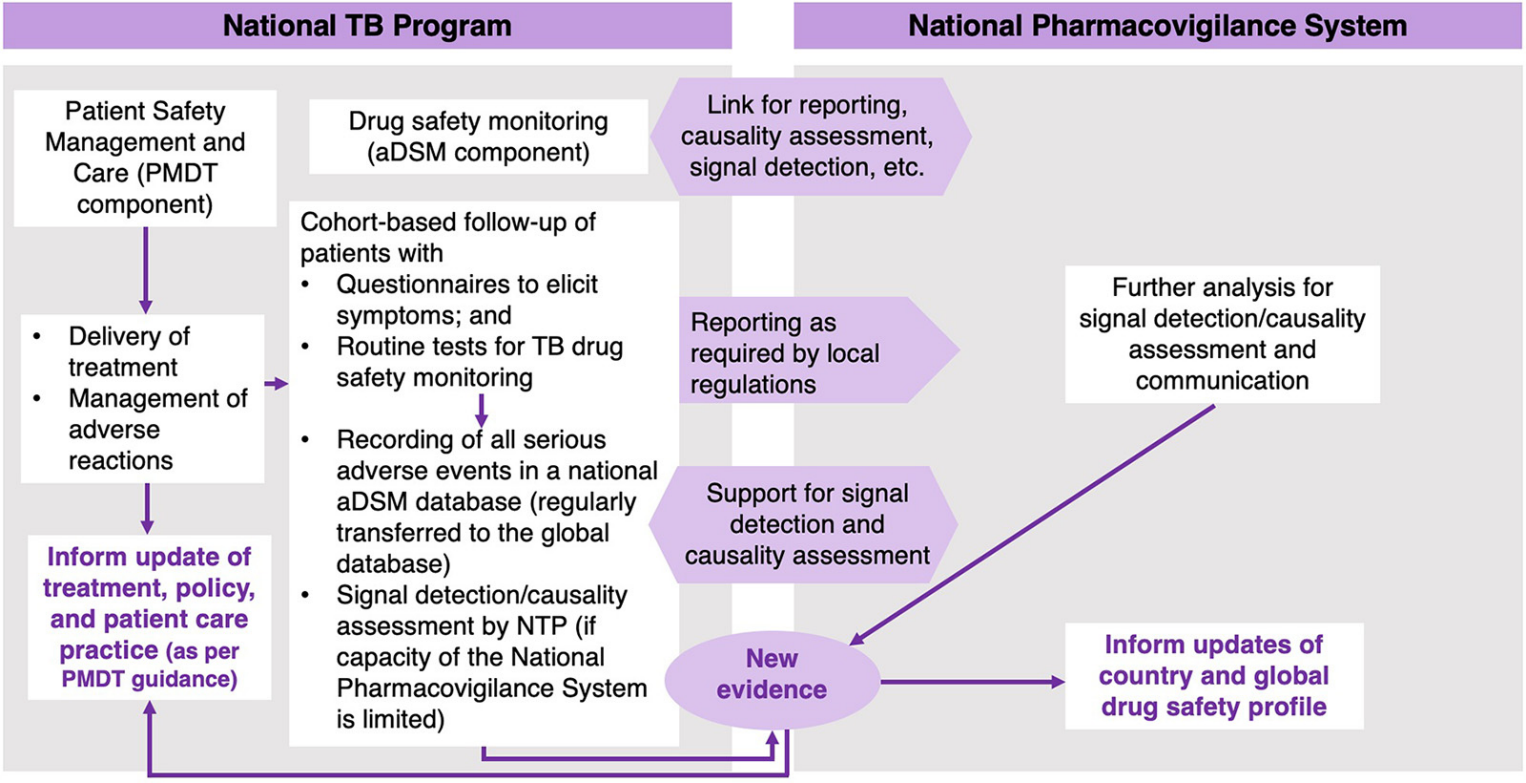
Generic Model of aDSM Within Drug-Safety Structures at the National Level Abbreviations: aDSM, active drug safety monitoring and management; PMDT, programmatic management of drug resistant TB; NTP, national TB program. Adapted from the World Health Organization Framework for aDSM Implementation.^22^

The WHO recommends that countries follow 8 steps to develop and integrate aDSM for the introduction of novel TB drugs and regimens (Box).^22^ In 2016, the WHO launched the global aDSM database to collect a standard set of variables including anonymized individual-level patient data on serious adverse events.^24^ aDSM, as recommended by the WHO, is being implemented in countries with a relatively high burden of rifampicin-resistant/MDR-TB with the support of local and international partners.^25^^,^^26^ By the end of 2019, 24 high MDR-TB burden countries were systematically collecting data on adverse events in their TB information systems.^2^ However, several of these were implemented in response to specific projects and may not have been sustained.^27^ In 2020, the WHO team on Pharmacovigilance; the Global TB Programme; and TDR, the Special Program for Research and Training in Tropical Diseases assessed the global aDSM database and decided to archive and store the data. However, they recommended that countries continue active monitoring following the WHO aDSM framework. aDSM within national TB programs must be well coordinated with the national pharmacovigilance systems (Figure 1).

BOX **World Health Organization's 8 Key Steps to Introduce Active Drug Safety Management^22^**

1. Create a national coordinating mechanism.
2. Develop a plan for active drug safety management (aDSM).
3. Define management and supervision roles and responsibilities.
4. Create standard data collection materials.
5. Train staff for collection of data.
6. Define schedules and routes for data collection and reporting.
7. Consolidate aDSM data electronically.
8. Develop (or use existing) capacity for signal detection and causality assessment.

An important component of aDSM is causality assessment, which is used to estimate the strength of the relationship between drug exposure and occurrence of ADRs.^28^ As in Figure 1, causality assessment can be performed within the national TB program or the national pharmacovigilance system and generally considers 4 criteria:

1. Association in time (and place) between the drug administration and the event
2. Pharmacology and pharmacokinetics, including previous knowledge of side effects, features of the event, site of reaction, dose-response effects (dose reduction and effect of reexposure), and known actions of the drug
3. Medical plausibility, including characteristic signs and symptoms, laboratory tests, and pathological findings
4. Likelihood or exclusion of other causes^23^

Other components of aDSM systems include data collection forms, pharmacovigilance standard operating procedures, forms for active monitoring and management of MDR-TB patients using clinical and laboratory assessment, coordinating mechanisms, and health worker training and ongoing mentoring. In 8 countries, FHI 360 supported the introduction and strengthening of national aDSM systems (the Supplement contains additional details of these efforts).^29^

## RATIONALE FOR ESTABLISHING AN ADSM-LIKE SYSTEM FOR ART MONITORING: ACTIVE TOXICITY SURVEILLANCE OF ART

As of June 2020, 26 of 38 million PLHIV (68%) worldwide were accessing ART.^1^ Increasing levels of pretreatment resistance to non-nucleoside reverse-transcriptase inhibitors (NNRTI) in LMICs is accelerating the need for expanded access to alternative non-NNRTI ARVs.^30^ In 2019, the WHO updated ART treatment recommendations to include the transition to integrase strand inhibitor-based ART as a priority, with a focus on the drug dolutegravir as an alternative to NNRTI ARVs.^9^ With the availability of a generic fixed-dose combination of dolutegravir with tenofovir and lamivudine and recent price reductions, many countries are now transitioning to the tenofovir, lamivudine, and dolutegravir combination, which has implications on the supply chain, provider preparedness, and the need for attention to possible adverse events or ADRs.^31^ Although several studies in children and adults conclude that routine toxicity monitoring is neither clinically required nor cost-effective, these findings were based on older regimens.^32^^,^^33^ Although, it is anticipated that newer regimens are actually less toxic than previous ones, it is important for programs to be vigilant to monitor for unexpected or previously unreported ADRs.

When novel agents or regimens such as ARVs are introduced, new unanticipated toxicities may arise, which were either not observed in clinical trials or their impact not fully understood before widespread scale-up. Due to the lack of real-world experience with integrase inhibitors on a large scale, in 2017, the WHO recommended that while countries transition to new ART regimens, a combination of active toxicity surveillance approaches with enhanced monitoring should be implemented in addition to routine toxicity monitoring.^9^^,^^34^ Recent data suggest a potential risk of weight gain associated with dolutegravir, with or without tenofovir alafenamide, which could affect health outcomes related to noncommunicable obesity-related diseases. Active toxicity monitoring is encouraged as these drugs are introduced and scaled up nationwide and globally.^35^

WHO recommended that while countries transition to new ART regimens, a combination of active toxicity surveillance approaches with enhanced monitoring should be implemented in addition to routine toxicity monitoring.

The WHO describes active toxicity monitoring as^31^:

*a system in which active measures are taken to detect the presence or absence of ADRs occurring during or after exposure to a pharmaceutical product.*

ADRs may be detected by interviewing patients, performing specific investigations, or by screening patients' medical records. The WHO currently recommends a combination of approaches to monitor ARV toxicity and promote patient safety, including active and routine toxicity monitoring.^34^^,^^36^^,^^37^ Active toxicity monitoring includes reporting on management, seriousness, and outcome of ADRs (Table 1).^31^ Some of the major known antiretroviral toxicities are included in Table 2.^38^

**TABLE 1.** Data and Specificity Captured by Active Toxicity Monitoring Versus Routine Toxicity Monitoring^a^

Data Element	Routine Toxicity Monitoring	Active Toxicity Monitoring
Management of adverse drug reactions	Partially (drug substitutions resulting from toxicity captured)	Yes
Seriousness of adverse drug reactions	No	Yes
Outcome of adverse drug reactions (resolved, requires, or prolongs hospitalization, disability, death, etc.)	No	Yes

a Described in the *World Health Organization Implementation Tool for Monitoring the Toxicity of New Antiretroviral and Antiviral Medicines in HIV and Viral Hepatitis Programmes*.^31^

**TABLE 2.** Major Antiretroviral Toxicities Described in the WHO Consolidated Guidelines on the Use of Antiretroviral Drugs for Treating and Prevention HIV Infection 2016^38^

Antiretroviral Drug	Toxicity
Abacavir	Hypersensitivity reaction
Atazanavir/r	Electrocardiographic abnormalities (PR and QRS interval prolongation) Indirect hyperbilirubinemia (clinical jaundice) Nephrolithiasis
Zidovudine	Severe anemia, neutropenia Lactic acidosis or severe hepatomegaly with steatosis Lipoatrophy Lipodystrophy Myopathy
Darunavir/r	Hepatotoxicity Severe skin and hypersensitivity reactions
Dolutegravir	Hepatotoxicity Hypersensitivity reactions
Efavirenz	Persistent central nervous system toxicity (such as dizziness, insomnia, abnormal dreams) or mental symptoms (anxiety, depression, mental confusion) Convulsions Hepatotoxicity Severe skin and hypersensitivity reactions Gynecomastia Severe skin and hypersensitivity reactions
Lopinavir/r	Electrocardiographic abnormalities (PR and QRS interval prolongation, torsades de pointes) Hepatotoxicity Pancreatitis Dyslipidemia Diarrhea
Nevirapine	Hepatotoxicity Severe skin rash and hypersensitivity reaction, including Stevens-Johnson syndrome
Raltegravir	Rhabdomyolysis, myopathy, myalgia Hepatitis and hepatic failure Severe skin rash and hypersensitivity reaction
Tenofovir Disoproxil Fumarate	Chronic kidney disease Acute kidney injury and Fanconi syndrome Decreases in bone mineral density Lactic acidosis or severe hepatomegaly with steatosis

**FIGURE 2:**
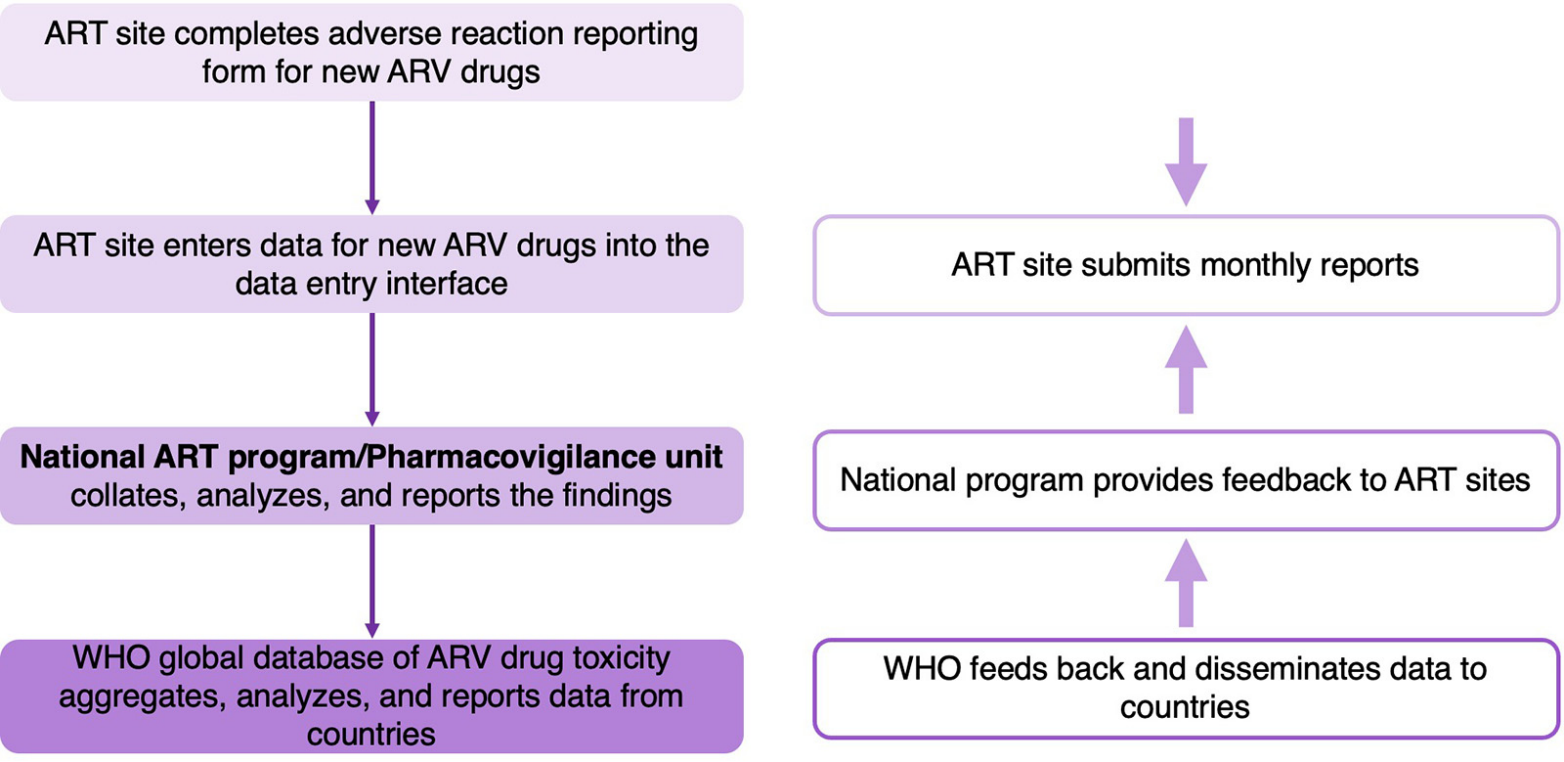
Data Collection and Reporting Process for Active Toxicity Monitoring for New ARV Drugs Abbreviations: ART, antiretroviral therapy; ARV, antiretroviral; WHO, World Health Organization. Adapted from the *World Health Organization Implementation Tool for Monitoring the Toxicity of New Antiretroviral and Antiviral Medicines in HIV and Viral Hepatitis Programmes*.^31^

Active toxicity monitoring of new ARVs should be linked to existing pharmacovigilance systems to complement routine monitoring and capture treatment-limiting ARV toxicities.^31^^,^^34^ Programs should monitor patients' body weight, body mass index, risk factors, metabolic comorbidities, cardiovascular disease, and pregnancy outcomes throughout treatment.^39^ Furthermore, national data can feed into the WHO global ARV active toxicity monitoring database for surveillance of novel ARVs to aggregate safety data collected at national levels and generate more rapid evidence on safety profiles, address gaps in safety data, and enhance detection of toxicity signals.^40^ WHO recommends the approach outlined in Figure 2 for bidirectional flow of information from ART sites to WHO database and vice versa. Countries are invited to contribute data in parallel while reporting to the Uppsala Monitoring Center database. Electronic database systems simplify processes to avoid excessive burden on country systems. Several dolutegravir early-adopter countries have already developed and implemented HIV-specific active toxicity monitoring systems, including Nigeria, Uganda, Botswana, Brazil, and Kenya.^31^ However, we propose that LMICs that have not yet invested in HIV active toxicity monitoring but have systems established for aDSM of TB medicines should consider building on what has been established for MDR-TB. Several countries fall into this category including Indonesia, Vietnam, and Cambodia. The Philippines is currently in the process of adopting such an approach. The aDSM infrastructure can be adapted for inclusion of HIV drugs for simplicity, economy of effort, and maximizing the impact of previous investments.

## RATIONALE FOR INTEGRATING ADSM INTO HIV PROGRAMMING AS A TB/HIV COLLABORATIVE ACTIVITY

The WHO recommends a package of collaborative TB/HIV activities (Table 3) to establish and strengthen collaboration and joint management between HIV and TB programs and to reduce the burden of TB among PLHIV and of HIV among those with confirmed or presumptive TB.^41^ These activities could be expanded upon to include elements of ADR monitoring to benefit both HIV and TB programs and to enhance quality of care for those living with HIV, TB, or both. For instance, Table 3, Section A, component A1 regarding establishing a coordinating body could include aDSM/active toxicity monitoring with joint systems development, strengthening, learning opportunities, and supervision.

**TABLE 3.** World Health Organization-Recommended Collaborative TB/HIV Activities^a^

**A. Establish and strengthen the mechanisms for delivering integrated TB and HIV services**
A.1. Set up and strengthen a coordinating body for collaborative TB/HIV activities functional at all levels.
A.2. Determine HIV prevalence among TB patients and TB prevalence among PLHIV.
A.3. Carry out joint TB/HIV planning to integrate the delivery of TB and HIV services.
A.4. Monitor and evaluate collaborative TB/HIV activities.
**B. Reduce the burden of TB in PLHIV and initiate early ART (the Three I's for HIV/TB)**
B.1. Intensify TB case-finding and ensure high quality antituberculosis treatment.
B.2. Initiate TB prevention with isoniazid preventive therapy and early antiretroviral therapy.
B.3. Ensure control of TB infection in health care facilities and congregate settings.
**C. Reduce the burden of HIV in patients with presumptive and diagnosed TB**
C.1. Provide HIV testing and counseling to patients with presumptive and diagnosed TB.
C.2. Provide HIV prevention interventions for patients with presumptive and diagnosed TB.
C.3. Provide cotrimoxazole preventive therapy for TB patients living with HIV.
C.4. Ensure HIV prevention interventions, treatment, and care for TB patients living with HIV.
C.5. Provide antiretroviral therapy for TB patients living with HIV.

Abbreviation: PLHIV, people living with HIV.

a Adapted from the WHO policy on collaborative TB/HIV activities.^41^

### Systematic Monitoring and Management of ADRs

Programs should consider a collaborative approach between TB and HIV programs to actively monitor ADRs. One critical reason is that patients who are coinfected with TB and HIV should take TB medicines and ARVs concurrently, according to guidelines based on clinical trial data showing better outcomes for those with TB and HIV who receive rapid TB treatment and ART.^38^^,^^42^ Furthermore, at the programmatic level, as treatment and care services for both diseases are decentralized in a differentiated and patient-centered approach, often the same primary care providers are managing both TB treatment and ART, for both coinfected as well as mono-infected patients. Thus, frontline clinicians are most likely to encounter ADRs and should be a-dept at ADR monitoring and management for TB and HIV. The Uppsala Monitoring Centre has an approved standard ADR reporting form which countries can make available in paper-based format or as part of electronic medical record systems across public and private health facilities to document ADRs. This first level of reporting, which then feeds into electronic databases such as the Pharmacovigilance Monitoring System^43^ or the Uppsala Monitoring Centre developed VigiFlow^44^ for further upstream reporting. These electronic systems can operate across diseases and have automated causality analysis and reporting systems to reduce the burden on health workers. Additional human resources may be required at the public health/disease control division of the ministry of health with a pharmacovigilance officer or data manager to support reporting across disease entities. LMICs should aim to develop a single, streamlined system for detection, monitoring, management, and recording of ADRs, with a comprehensive training program that simultaneously covers both diseases for front-line clinicians and other health workers at the community level.

Programs should consider a collaborative approach between TB and HIV programs to actively monitor ADRs.

### Collaborative Funding Streams

Frequently, TB and HIV funding streams, and thus program implementation, run in vertical silos. Without coordination, parallel systems for monitoring treatment response are often developed requiring 2 sets of protocols, forms, trainings, and monitoring processes. There are 3 major components of aDSM to consider regarding costs: clinical management, laboratory components, and reporting systems. The first 2 components are necessary to provide quality care for both TB and HIV, and no additional investment is required in an integrated approach. Existing electronic information systems may already function as multidisease platforms with automated causality analysis systems and reporting systems. If 1 disease program has already invested in establishing an information system, there is no need for another disease program to also do so. The only additional resource required is human resources, which can be pooled under the management of public health control divisions of ministries of health through placement of a pharmacovigilance officer or data manager to support all diseases. Previous donor investments to establish aDSM systems for a single disease, such as TB, can then be leveraged to enable patients requiring treatment for other diseases to also benefit. For more efficient use of resources and streamlined health service operations, we propose that in countries where aDSM for MDR-TB is already available and functioning, systems, tools, and experienced staff could be leveraged to support active toxicity monitoring of new ARVs with adaptation as needed.

We propose that in countries where aDSM for MDR-TB is already available and functioning, systems, tools, and experienced staff could be leveraged to support active toxicity monitoring of new ARVs with adaptation as needed.

### Causality Assessment

Causality assessment is another avenue for potential collaboration. A TB/HIV causality assessment core committee could convene where cases involving TB medicines and ARVs could be discussed in the same forum. The committee could conduct formal causality assessments together with members from the national drug regulatory authority or separately directing information up to the national program authority (i.e., National TB Program or National AIDS Program). To ensure accurate and up-to-date information sharing, initial and refresher causality assessment training of the committee and others involved in the process could benefit both TB and HIV programs.

### Implementation Issues

Looking at a specific example in Papua New Guinea, aDSM scale-up and extension from TB systems to include HIV was successfully implemented in the country's National Capital District province, with further scale-up planned to reach national coverage in 2022. This coincides with the successful transition of all adult PLHIV to the tenofovir-lamivudine-dolutegravir regimen and with the recent introduction of pediatric dolutegravir. Longitudinal toxicity monitoring requires more extensive efforts, but electronic record management systems are helping to support the process. A significant challenge to implementation in this setting has been the chronic shortage of human resources with overstretched care service providers. Data recording and reporting are often a low priority, and few clinicians are available to support active monitoring and managing clients with ADRs. In addition, the country suffers from limited laboratory capacity, which is an essential component of toxicity monitoring.

## CONCLUSION

New tools including therapeutics are needed to achieve global HIV epidemic control as well as TB elimination. After a relatively long gap in the development and approval of new therapeutics for TB, several new regimens for treatment and prevention are currently being introduced globally. Widespread transition to better ART regimens is also underway. Mechanisms to ensure safe treatment scale-up for both diseases are critical. Although pharmacovigilance systems have been established in most LMICs, they are frequently limited to passive surveillance and are often suboptimally functioning. A more active approach to managing toxicity of new TB and HIV medicines and regimens is required yet is nascent or nonexistent in many high-burden TB and HIV countries. Several national TB programs have successfully established aDSM systems to monitor and manage toxicities from MDR-TB medicines, and elements of this work can be harnessed to support HIV program goals of reducing ART toxicity.

We propose a collaborative approach tailored to specific country contexts where previous aDSM development for TB and strengthening of pharmacovigilance systems are leveraged to support the introduction of active toxicity monitoring for HIV programs. ADRs from both programs can be detected, reported, and managed through streamlined, integrated and resource-efficient approaches rather than developing siloed systems. This could also expand to treatment of hepatitis, other infectious diseases, and even noncommunicable diseases depending on the setting and need. A multidisease causality assessment committee would bring expertise to decentralized systems and ultimately reach more patients. Further efforts to link active toxicity monitoring and aDSM to national pharmacovigilance systems should ultimately feed into global databases to contribute to more rapid accumulation of evidence to help populations achieve maximum benefit from novel drugs and regimens while ensuring close monitoring of safety issues.

## Supplementary Material

GHSP-D-21-00595-supplement.docx

## Notes

**Peer Reviewed**

**First published online:** March 17, 2022.
