# Supplementary material for: Leveraging Experience From Active TB Drug-Safety Monitoring and Management for Monitoring Active Antiretroviral Toxicity
Source: Glob Health Sci Pract. 2022 Apr 28;10(2):e2100595. doi: 10.9745/GHSP-D-21-00595 (PMC9053160; doi:10.9745/GHSP-D-21-00595)
Supplement: GHSP-D-21-00595-supplement.docx [file GHSP-D-21-00595-supplement.docx]

**Examples of FHI360 aDSM Support**

In Papua New Guinea, Myanmar, China, Thailand, Cambodia, Zambia, Mozambique, and the Philippines, FHI 360 has supported the introduction and strengthening of national aDSM systems through development of coordinating mechanisms, roadmaps, implementation plans, and protocols as well as providing training, ongoing mentoring, and technical assistance.

For instance, in Papua New Guinea, the organization assisted the National Department of Health to develop systems for TB aDSM and pharmacovigilance through the USAID Challenge TB and USAID Control and Prevention of TB projects. FHI 360 developed the pharmacovigilance standard operating procedures and forms for active monitoring and management of MDR-TB patients using clinical and laboratory assessment. These forms were then adapted for the country’s pharmacovigilance system at large.^29^ Adverse events reporting forms and systems were also developed for communication to the National programmatic management of drug resistant TB/aDSM core group and the Causality Assessment Committee. FHI 360 worked closely with relevant stakeholders to expand aDSM and pharmacovigilance at the national level using regional Medicines and Therapeutics Committees, helping to ensure sustainability. Finally, with FHI 360’s support, Papua New Guinea achieved full membership to the Uppsala Monitoring Center that oversees technical and operational aspects of the WHO Program for International Drug Monitoring.
